# HIF-3α/PPAR-γ Regulates Hypoxia Tolerance by Altering Glycolysis and Lipid Synthesis in Blunt Snout Bream (*Megalobrama amblycephala*)

**DOI:** 10.3390/ijms26062613

**Published:** 2025-03-14

**Authors:** Minggui Jiang, Jing Huang, Xing Guo, Wen Fu, Liangyue Peng, Yang Wang, Wenbin Liu, Jinhui Liu, Li Zhou, Yamei Xiao

**Affiliations:** 1 College of Life Sciences, Hunan Normal University, Changsha 410081, China; jmg202310140286@hunnu.edu.cn (M.J.); 202410140333@hunnu.edu.cn (J.H.); guoxing@hunnu.edu.cn (X.G.); fuwen@hunnu.edu.cn (W.F.); ply@hunnu.edu.cn (L.P.); wenbinliu@hunnu.edu.cn (W.L.); jinhuiliu0731@hunnu.edu.cn (J.L.); 2 Engineering Research Center of Polyploid Fish Reproduction and Breeding of the State Education Ministry, Changsha 410081, China; 3 State Key Laboratory of Freshwater Ecology and Biotechnology, Hubei Hongshan Laboratory, The Innovation Academy of Seed Design, Institute of Hydrobiology, Chinese Academy of Sciences, Wuhan 430072, China; wangyang@ihb.ac.cn (Y.W.); zhouli@ihb.ac.cn (L.Z.)

**Keywords:** hypoxia tolerance, HIF/PPAR-γ signaling pathway, energy metabolism, blunt snout bream

## Abstract

Hypoxic stress causes cell damage and serious diseases in organisms, especially in aquatic animals. It is important to elucidate the changes in metabolic function caused by hypoxia and the mechanisms underlying these changes. This study focuses on the low oxygen tolerance feature of a new blunt snout bream strain (GBSBF1). Our data show that GBSBF1 has a different lipid and carbohydrate metabolism pattern than wild-type bream, with altering glycolysis and lipid synthesis. In GBSBF1, the expression levels of *phd2* and *vhl* genes are significantly decreased, while the activation of HIF-3α protein is observed to have risen significantly. The results indicate that enhanced HIF-3α can positively regulate gpd1ab and gpam through PPAR-γ, which increases glucose metabolism and reduces lipolysis of GBSBF1. This research is beneficial for creating new aquaculture strains with low oxygen tolerance traits.

## 1. Introduction

As the core matter of cell metabolism and function, oxygen (O_2_) plays an important part in life activity. Fluctuations in dissolved oxygen levels are typical for species in the aquatic environment. The growth, metabolism, and reproductive capacities of aquatic species can be negatively affected by hypoxic stress. It often leads to stress responses and high mortality rates of aquatic organisms, seriously threatening the stability of aquatic ecosystems, especially in aquaculture [1,2,3,4]. Therefore, the discovery of the molecular regulation mechanism of low oxygen adaptation not only aids in understanding the normal physiological functions of fish, but also promotes the prevention and treatment of related diseases in aquaculture through an understanding of hypoxic diseases.

Hypoxia-inducible factors, or HIFs, which sense the hypoxic environment within cells, stimulate the metabolism of cells, modulate the proliferation of the cells, and regulate inflammatory responses and other functions, are the main pathways for cellular response to hypoxic conditions [5]. HIFs are categorized as a member of the PAS (Per-ARNTSim) family and are composed of an oxygen-sensitive α subunit and one constitutively expressed β subunit. HIF-α proteins (HIF-1α, HIF-2α, and HIF-3α) and HIF-β proteins can remain active in the form of heterodimers [6]. PHD2 and pVHL are key proteins in the HIF signaling pathway [7,8]. Two proline sites are hydroxylated by oxygen-dependent proline hydroxylases (PHDs) in HIF-α’s oxygen-dependent degradation domain [9]. Hydroxylated HIF-α interacts with von Hippel–Lindau (VHL), promoting the ubiquitin-proteasome degradation of HIF-α [10]. The proteasome quickly ubiquitinates and destroys HIF-α proteins under normal oxygen conditions [11]. The phylogenetic analysis of HIF-α genes in fish demonstrated that they possess orthologs of the three genes found in mammals [12].

*Megalobrama amblycephala* (blunt snout bream, BSB) is an economically important fish with tender and delicious meat. However, as an oxygen-sensitive species, BSB often dies extremely easily when dissolved oxygen (DO) levels fall below 0.5 mg/L [13]. In our early work, eggs from wild-type BSB were used to successfully breed artificial gynogenesis BSB (GBSB), following activation by UV-irradiated sperm from red crucian carp (*Carassius auratus red var*), and treatment doubling the number of chromosomes was performed. GBSB consist entirely of a female population and exhibit hypoxia tolerance [14]. In this study, we used GBSB as the female parent to breed with wild-type male BSB, and obtained mass-produced filial generation (GBSBF1). Compared to wild-type BSB, GBSBF1 still retained excellent hypoxia tolerance, which is not only a potential new breeding target, but also provides sufficient research materials for the molecular mechanisms of fish tolerance to low oxygen levels. We further analyzed the possible low oxygen tolerance feature of GBSBF1 through biochemical testing, pathological detection, and transcriptomics analysis. This research aimed to elucidate the molecular mechanisms underlying fish tolerance to low oxygen levels, and helped to develop excellent strains with strong hypoxia tolerance in fish.

## 2. Result

### 2.1. GBSBF1 Is a New Strain of BSB with Improved Hypoxia Tolerance

**Figure 1:**
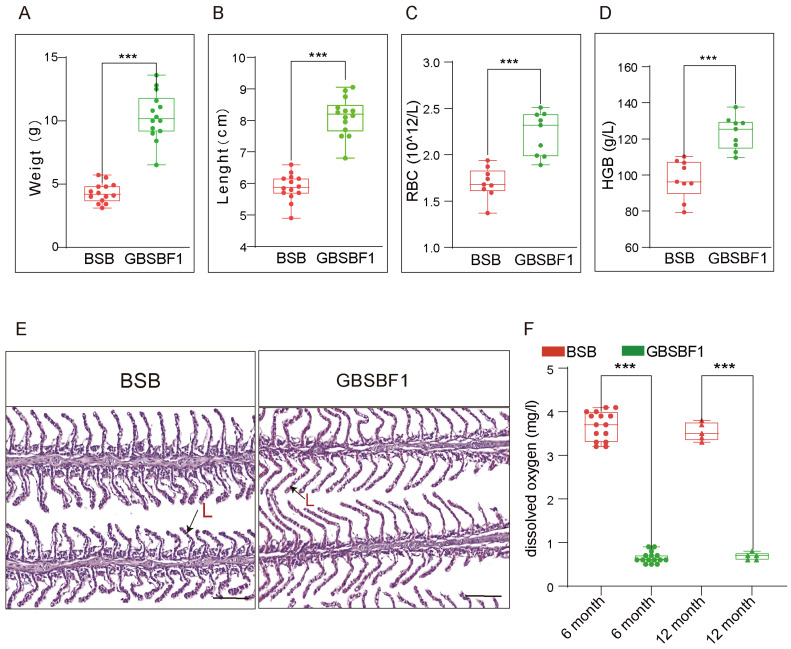
GBSBF1 is a new hypoxia-tolerant strain of Megalobrama amblycephala with a faster growth rate. (**A**) Comparison of weights between GBSBF1 and BSB at 6 months old (*n* = 14, SD; ***, *p* < 0.001). (**B**) Comparison of lengths between GBSBF1 and BSB at 6 months old (*n* = 14, SD; ***, *p* < 0.001). (**C**) Analysis of the red blood cell count (RBC) of BSB and GBSBF1 (*n* = 3, SD;***, *p* < 0.001). (**D**) Analysis of the hemoglobin content (HGB) of BSB and GBSBF1 (*n* = 3, SD; ***, *p* < 0.001). (**E**) HE staining of the gills of BSB and GBSBF1. L represents the gill lamellae, bar = 100 μm. (**F**) Analysis of the hypoxia tolerance of BSB and GBSBF1 at different stages. Fish at 6 months (*n* = 15) and 12 months (*n* = 5) under the same treatment conditions were sampled (SD; ***, *p* < 0.001).

We obtained the progeny GBSBF1 by backcrossing GBSB with wild-type BSB. Compared with BSB, GBSBF1 grows at a faster rate (Figure S1). Under the same feeding conditions, both the weight and length of 6-month-old GBSBF1 are significantly higher than those of ordinary BSB (Figure 1A,B). Blood sample analysis revealed that GBSBF1 had significantly higher erythrocyte counts and hemoglobin levels compared to BSB (Figure 1C,D). Histological examination of the gills also showed distinct differences between GBSBF1 and BSB. GBSBF1 exhibited longer gill lamellae than those of BSB (Figure 1E). All these adaptations contributed to enhanced hypoxia tolerance in GBSBF1.

GBSB was charactered by its enhanced hypoxia tolerance compared to wild-type BSB [14]. To determine whether its filial generation, GBSBF1, has inherited this advantageous trait, we firstly conducted hypoxia stress experiments on 15 individuals each of BSB and GBSBF1, aged 6 months. The dissolved oxygen in the water was 0.66 ± 0.06 mg/L when GBSBF1 initially showed floating head, which was significantly lower than that when BSB first showed signs of floating to the water surface (3.69 ± 0.33 mg/L). Additionally, we also conducted hypoxia tolerance experiments on samples approximately 12 months old. Consistent with previous results, the DO in the water when 12-month-old GBSBF1 initially exhibited floating head was also significantly lower than that of the BSB of the same batch (0.68 ± 0.08 mg/L vs. 3.54 ± 0.21 mg/L, Figure 1F). These findings indicated that GBSBF1 had superior hypoxia tolerance traits compared to BSB.

### 2.2. The HIF Signaling Pathway Is Enhanced in GBSBF1

To reveal the molecular mechanism underlying the increased hypoxia tolerance of GBSBF1, we selected tissue (liver, heart, brain, and gills) from three BSB and GBSBF1 subjects, all maintained under identical conditions, for transcriptome sequencing. Principal component analysis (PCA) showed that the samples of the liver and heart of the two groups of fish had a good degree of discrimination (Figure S2A), while cluster analysis indicated that the RNA-seq data of the liver and gills were clustered together (Figure S2B). Based on the above, we selected the RNA-seq data of liver samples as the basis for subsequent analysis. Through RNA-seq analysis, a total of 2428 differentially expressed genes (DEGs) were identified. Among them, 1572 genes were upregulated and 856 genes were downregulated in GBSBF1 (Figure S2C).

**Figure 2:**
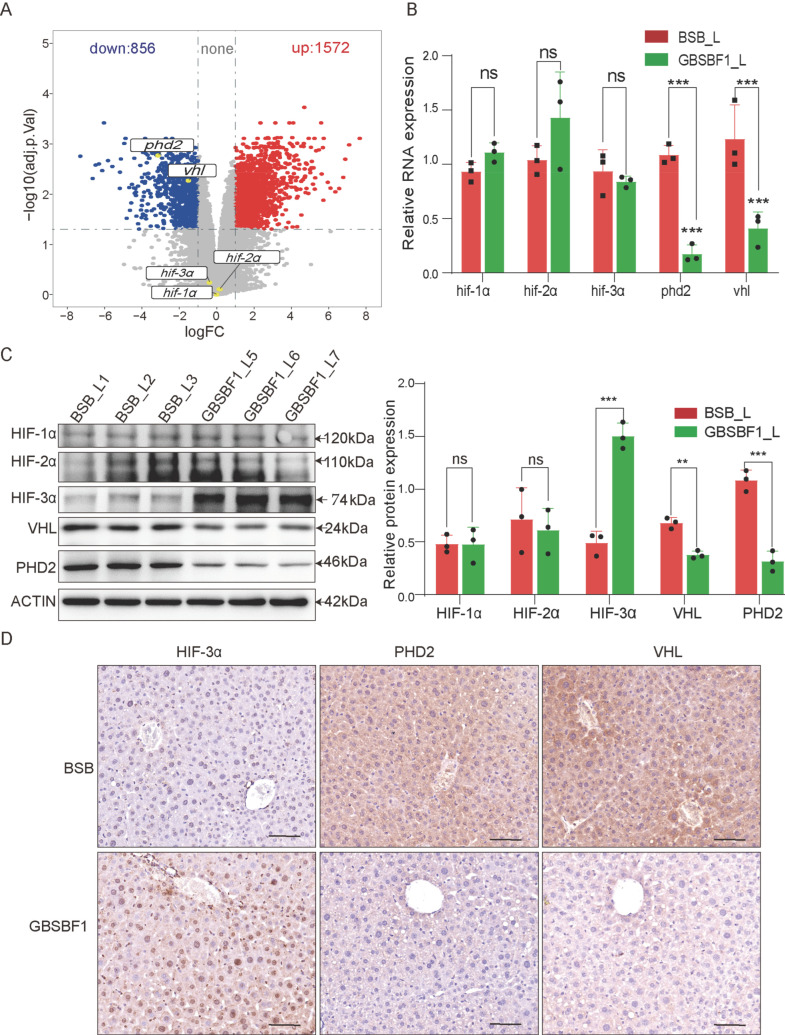
The HIF pathway is enhanced in GBSBF1. (**A**) Volcano plot analysis of RNA-seq data, in which the core genes of the HIF pathway, such as hif-1α, hif-2α, hif-3α, phd2, and vhl were specifically annotated. (**B**,**C**) The mRNA and protein expression levels of the core genes in the HIF signaling pathway were measured by qRT-PCR and Western blot (*n* = 3, SD; **, *p* < 0.01; ***, *p* < 0.001, and ns for no significant difference). (**D**) The protein expression of the core genes in the HIF signaling pathway in the liver by means of immunohistochemistry (IHC), bar = 100 μm.

Given that GBSBF1 exhibited greater hypoxia tolerance than BSB, we subsequently examined the expression of genes within the HIF signaling pathway, which is directly associated with hypoxia tolerance. Remarkably, the expressions of *phd*2 and *vhl* were dramatically reduced in GBSBF1, especially *phd*2, in spite of the fact that there was no significant difference in the transcriptional expression of *hif-α* (*hif-*1*α*, *hif-*2*α*, and *hif-*3*α*) between GBSBF1 and BSB (Figure 2A). This finding was corroborated by qRT-PCR and WB results (Figure 2B,C). In addition, our data showed that although the gene transcriptional level of *hif-α* does not change significantly, the amount of active HIF-3α protein that is visible increases (Figure 2C,D). These results suggested that under normoxic conditions, the reduced PHD2 and VHL in GBSBF1 likely lead to a reduction in the amount of degraded HIF-α, and the primary HIF-α isoform affected is HIF-3α. Consequently, the level of active HIF-3α increases, thereby activating the HIF signaling pathway.

### 2.3. GBSBF1 Has Different Lipid and Carbohydrate Metabolism Patterns Compared to BSB

**Figure 3:**
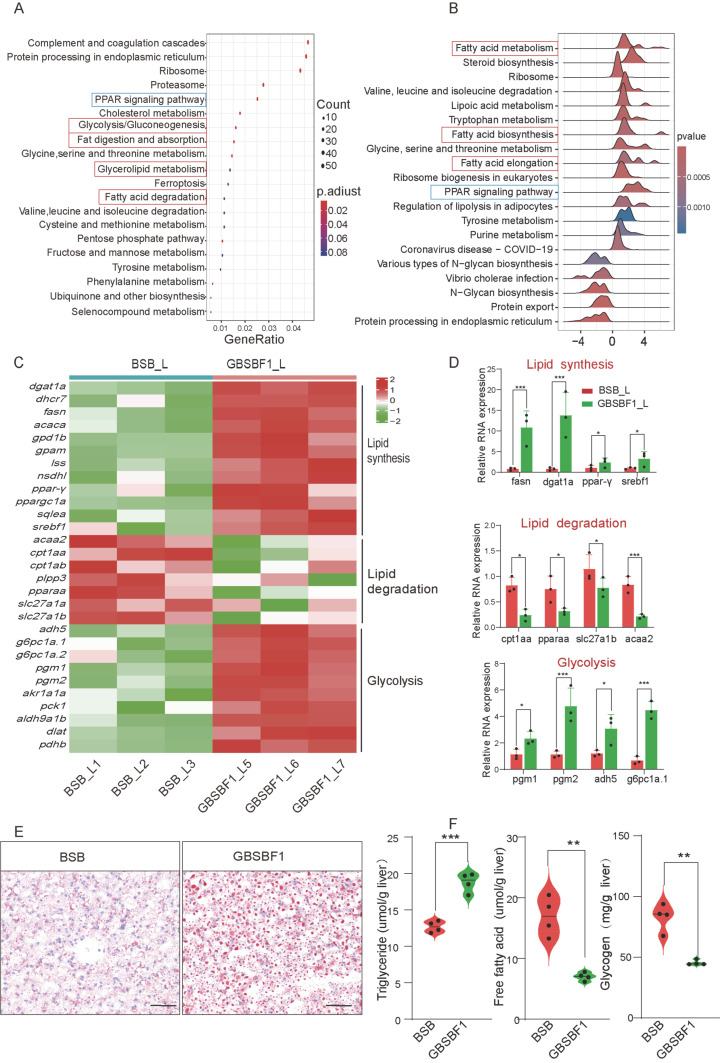
GBSBF1 has different lipid and carbohydrate metabolism patterns compared to BSB. (**A**) RNA-seq data were used to perform KEGG enrichment analysis of the differentially expressed genes, and the signal pathways related to lipid metabolism and glucose metabolism are framed (red). (**B**) Data from RNA-seq were analyzed using GSEA analysis and the signal pathways related to lipid metabolism and glucose metabolism are framed (red). (**C**) A portion of the core genes related to lipogenesis, lipolysis, and glycolysis are shown in the heatmap. (**D**) The mRNA expression levels of a portion of the core genes in connection to lipogenesis, lipolysis, and glycolysis were determined using qRT–PCR (*n* = 3, SD; *, *p* < 0.05; ***, *p* < 0.001). (**E**) Oil Red O staining of the livers of BSB and GBSBF1 was conducted, and the results showed that lipid droplets in the liver samples of GBSBF1 were significantly higher than that of BSB. bar = 50 μm. (**F**) Different levels of lipid and glucose metabolic products in the liver were detected by ELISA assay (n = 4, SD; **, *p* < 0.01; ***, *p* < 0.001).

Kyoto Encyclopedia of Genes and Genomes (KEGG) analysis indicated that the DEGs in the livers of BSB and GBSBF1 were enriched in metabolic pathways, including glycolysis and lipid metabolism (Figure 3A). Gene Set Enrichment Analysis (GSEA) also obtained conclusions similar to those of KEGG (Figure 3B). GSEA analysis of Gene Ontology (GO) related to biological processes (BP) showed significant differences in glucose metabolism and lipid metabolism pathways between the two groups (*p* < 0.05) (Figure S3A,B). It is worth noting that the PPAR pathway related to lipid metabolism was also enriched and showed significant differences (Figure S3C). This suggests differences in the mechanisms underlying energy metabolism in the livers between GBSBF1 and BSB. Therefore, we focused our investigation on genes associated with lipogenesis, lipolysis, glycolysis, and other related pathways. Heat map analysis of the transcriptome showed that, compared to BSB, the expression of genes related to lipid degradation decreased in GBSBF1, while the expression of genes related to lipid synthesis (including ppar-γ) and glycolysis increased (Figure 3C). The experimental results of qRT-PCR confirmed the changes in genes identified in the transcriptome analysis (Figure 3D).

Next, we detected the products of lipid metabolism and glucose metabolism. The results of Oil Red O staining showed that, compared with BSB, the number of lipid droplets in the liver of GBSBF1 increased significantly (Figure 3E). ELISA assay also showed that products related to lipolysis (e.g., free fatty acid (FFA)) decreased, products related to lipogenesis (e.g., triglyceride (TG)) increased, and glycogen decreased (Figure 3F). These results indicate that GBSBF1 has more lipid synthesis and glycolysis, while there is more lipolysis in BSB.

### 2.4. HIF-3α Promotes Glycolysis and Lipid Synthesis by Positively Regulating PPAR-γ in GBSBF1

**Figure 4:**
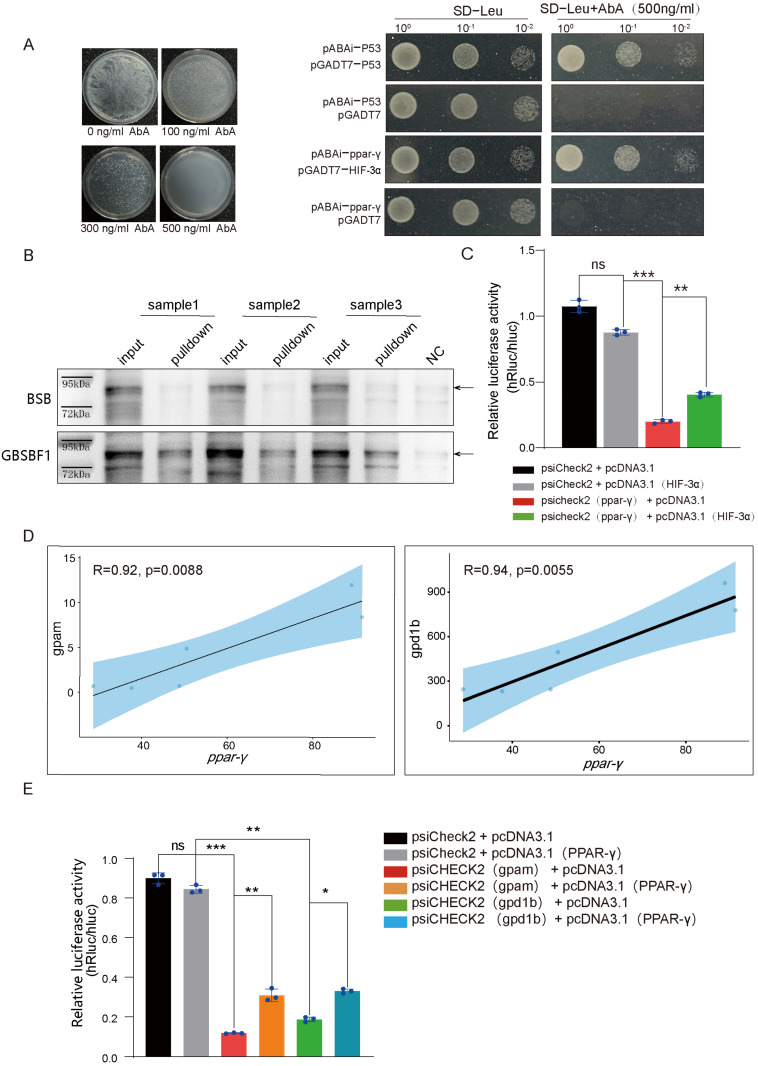
HIF-3α/PPAR-γ promotes lipid synthesis and glycolysis by positively regulating gpd1b/gpam in GBSBF1. (**A**) The yeast one-hybrid assay demonstrated that the HIF-3α protein of Megalobrama amblycephala could bind to the PPAR-γ promoter. Among them, the left-hand figure shows the screening results of different concentrations of AbA, while the right-hand figure shows the growth of yeast under conditions with and without the highest inhibitory concentration of AbA. (**B**) The DNA pull-down assay demonstrated that more HIF-3α could bind to the promoter of PPAR-γ in GBSBF1. (**C**) Dual luciferase assay indicates that HIF-3α promotes the transcription of downstream genes of ppar-γ (*n* = 3, SD; **, *p* < 0.01; ***, *p* < 0.001, and ns for no significant difference). (**D**) Correlation analysis of RNA-seq data shows the expression correlation between ppar-γ and its downstream target genes gpd1b and gpam. (**E**) Dual luciferase assay demonstrates that PPAR-γ promotes the transcription of downstream genes of gpam and gpd1b (*n* = 3, SD; *, *p* < 0.05; **, *p* < 0.01; ***, *p* < 0.001, and ns for no significant difference).

Studies have reported that in mammals, PPAR-γ is a key mediator of glycolysis and lipid synthesis metabolism and is a direct target of HIF-1α [15,16]. Furthermore, RNA-seq showed that the PPAR signaling pathway is one of the pathways with the most significant changes in the livers of BSB and GBSBF1 (Figure S3C, and marked with a blue frame in Figure 2A,B). We aimed to investigate whether PPAR-γ is regulated by HIF-3α in blunt snout bream. We first performed a yeast one-hybrid assay, and the results showed that the HIF-3α protein of Megalobrama amblycephala could bind to the promoter region of its *ppar-γ* gene (Figure 4A). Subsequently, we conducted a DNA pull-down assay using liver samples from BSB and GBSBF1. The results indicated that in GBSBF1, HIF-3α could indeed bind to the promoter of PPAR-γ more effectively (Figure 4B). Moreover, the dual luciferase assay further demonstrated that a high level of HIF-3α could promote the transcriptional level of PPAR-γ by binding to its promoter (Figure 4C).

Studies have shown that PPAR-γ can regulate glycolysis and lipid synthesis through target genes such as GPD1 (Glycerol-3-phosphate Dehydrogenase, the ortholog of gpd1b in Megalobrama amblycephala) and GPAT (Glycerol Phosphate Acyltransferase, the ortholog of gpam in Megalobrama amblycephala) [17,18]. RNA-seq data showed that the expression of *ppar-γ* correlated well with *gpd1b* and *gpam* (Figure 4D). Furthermore, as a transcriptional factor, PPAR-γ can positively regulate the expressions of *gpd1b* and *gpam* in GBSBF1 (Figure 4E). Therefore, PPAR-γ can serve as an important target of active HIF-3α, increasing lipid synthesis and glycolysis in GBSBF1, inhibiting lipolysis, and enhancing the hypoxia tolerance of GBSBF1 by regulating the key genes *gpd1b* and *gpam* during the transformation of glycolipid metabolism.

## 3. Materials and Methods

### 3.1. Ethics Statement

Fish-based experimental procedures received approval from the Institutional Animal Care and Use Committee of Hunan Normal University. Procedures were carried out according to the regulations of the Administration of Affairs Concerning Experimental Animals for the Science and Technology Bureau of China.

### 3.2. Fish

BSB (wild-type BSB), GBSB (gynogenetic BSB) and GBSBF1 were maintained at the State Key Laboratory of Developmental Biology of Freshwater Fish, College of Life Sciences, Hunan Normal University. Among them, GBSBF1 was produced by breeding with GBSB as the female parent and BSB as the male parent.

### 3.3. Hypoxia Treatment

In this study, hypoxia treatments were conducted on 6-month-old and 12-month-old fish, respectively. Fifteen individuals each of 6-month-old BSB and GBSBF1 were transferred separately into two 60 L tanks. Similarly, five individuals each of 12-month-old BSB and GBSBF1 were used for the experiment. These fish were accustomed to the same conditions for a week and fasted for three days before starting the experiment. Aeration equipment was employed to maintain the dissolved oxygen (DO) concentration in water at 6.0 ± 0.5 mg/L. By filling the tank with nitrogen, the DO was gradually reduced to simulate a natural hypoxic environment. In this study, the initial appearance of floating head was used as an indicator of hypoxia, and the DO level at which this occurred was measured with a DO meter (AM39, Munich, Germany). These experiments were repeated at least three times with different fish.

### 3.4. Physiological and Biochemical Analysis

Fish were anesthetized with 100 mg/L MS-222 (Sigma-Aldrich, St. Louis, MO, USA, E10521), and blood samples were collected from each fish using vacuum blood collection tubes. A total of nine wild-type BSB and nine GBSBF1 were sampled. These samples were analyzed using an XE 2100 hematology analyzer (Sysmex, Kobe, Japan), following the method described in a previously published paper [14].

Physiological and biochemical indexes were measured using liver samples from BSB and GBSBF1. All detection kits (triglycerides (A110-1-1), free fatty acids (A042-1-1), and glycogen (A043-1-1)) were purchased from Nanjing Jiancheng Bioengineering Institute (Nanjing, China), China, and the assays were performed according to the manufacturer’s instructions.

### 3.5. Hematoxylin–Eosin Staining

The gill and liver of BSB and GBSBF1 were first fixed in 4% paraformaldehyde fixative for 24 h. The samples were then dehydrated through a series of graded ethanol (70–100%), cleared with xylene, and embedded in paraffin. The paraffin-embedded sections were then washed with xylene and rehydrated through a graded ethanol series (100–70%). Subsequently, the sections were stained with hematoxylin and eosin (HE), and imaged using an optical microscope (Leica DM2000, Wetzlar, Germany).

### 3.6. Oil Red O Stainin

The liver samples from BSB and GBSBF1 were first fixed with 10% neutral formalin fixative (#Top0372, Biotopped, Beijing, China). Frozen sections were prepared from the fixed samples, stained with Oil Red O (Beyotime, Shanghai, China), and subsequently observed and photographed under a microscope.

### 3.7. qRT-PCR

Total RNA was extracted from livers of the same batch for transcriptome sequencing using Trizol reagent (Tiangen, Beijing, China), then the first strand of cDNA was produced using a reverse transcription kit (ThermoFisher, Waltham, MA, USA). qRT-PCR was performed using PerfectStart Green qPCR SuperMix (Trans, Beijing, China). The volume of each reaction was 10 μL, consisting of 5 μL SYBR-Green Master Mix, 0.2 μL forward primer, 0.2 μL reverse primer, 0.2 μL template cDNA, and 4.4 μL ddH2O. The number of thermal cycles was 40. The qPCR data were analyzed using the 2^−ΔΔCT^ method. All primers used in this study were shown in Table S1. The experiment was repeated three times.

### 3.8. Western Blotting

Total protein was extracted from liver specimens of the same batch used for transcriptome sequencing using RIPA lysis buffer (Beyotime, Shanghai, China). A BCA protein concentration assay kit (Beyotime, P0012) was used to measure the concentration of proteins. The samples were separated on 10% SDS-PAGE and transferred to a PVDF membrane (Millipore, Darmstadt, Germany). The membrane was blocked using 5% non-fat milk, and the primary antibody was incubated at 4 °C overnight. Protein were observed with BeyoECL Plus (Beyotime, P0018S) following an hour of incubation at room temperature with the matching HRP-conjugated secondary antibody. The Western blot was imaged with the Gel Doc XR system (Bio-rad, Hercules, CA, USA) and then analyzed using Image J. The expression of specific proteins was normalized to the internal reference protein β-actin. The information of the primary antibodies used in the Western blots was listed in Table S2.

### 3.9. Immunohistochemistry (IHC)

Antigen retrieval was performed on paraffin-embedded liver sections after they had been dewaxed and rehydrated. The slides were blocked with 10% normal goat serum and then incubated with the primary antibody overnight at 4 °C. Signal visualization was carried out using a mouse- and rabbit-specific HRP/AEC IHC detection kit (Servicebio, Wuhan, China). Table S2 lists the main antibodies for IHC.

### 3.10. Luciferase Assay

HepG2 cells (1 × 10^5^/well) were cultured for 24 h before transfection with plasmid DNA using LipoFiter (Hanbio, Shanghai, China), and transfection was carried out in 12-well plates. To detect the binding of transcription factor HIF-3α to the promoter of PPAR-γ, a dual luciferase detection system (psiCHECK2) was used. The promoter of *ppar-γ* of BSB replaced the SV40 promoter in psiCHECK2 to drive the expression of the Renilla luciferase gene, while the firefly luciferase served as a control. Meanwhile, an overexpression plasmid of HIF-3α of BSB (hypoxia-inducible factor-3α) was constructed and co-transfected into HepG2 cells to determine whether HIF-3α can regulate the transcriptional activity of *ppar-γ* in BSB. Similarly, the sv40 promoter in psiCHECK2 was substituted by the *gpd1b* or *gpam* promoter. These two plasmids were separately co-transfected with a PPAR-γ overexpression plasmid into HepG2 cells to determine whether PPAR-γ could regulate the transcriptional activities of *gpd1b* or *gpam*. The luciferase assay was carried out according to the manufacturer’s instructions (Promega, Madison, WI, USA). Results were gathered from at least three independent experiments.

### 3.11. DNA Pull-Down Assay

The DNA pull-down assay was performed according to the kit manufacturer’s instructions (BersinBio company, Guangzhou, China). First, fragments were designed and amplified against the PPAR-γ promoter region containing the HIF binding site (HRE), while DNA-specific probes were prepared and labeled with desulfobiotin. Streptavidin was coupled to magnetic beads and affinity-bound to desulfurized biotin. Nuclei extracts from liver samples of BSB and GBSBF1 were incubated with a magnetic bead DNA probe and eluted after washing and removing non-specifically bound protein molecules to obtain the DNA probe–protein, which was finally assayed by WB.

### 3.12. Yeast One-Hybrid Assay

The PPAR-γ promoter region was amplified and inserted into the bait vector pAbAi. After linearization, this construct was transformed into the Y1H Gold strain to generate the Y1H bait strain. To determine the highest inhibitory concentration of aureobasidin (AbA), URA media containing various concentrations of AbA were used for self-activation detection. The full cDNA of HIF-3α was inserted into the prey vector pGADT7. The bait and prey vectors were simultaneously transformed into yeast strains, and then screened on SD/-LEU plates containing the highest inhibitory concentration of AbA.

### 3.13. Transcriptome Sequencing

The transcriptome sequencing was performed by Wuhan Onemore-tech Co., Ltd. (Wuhan, China). The qualified libraries were sequenced on the DNBSEQ-T7RS platform. The genome of Megalobrama amblycephala (ASM1881202v1) from the NCBI database was used as the reference genome. The differentially expressed genes (DEGs) between BSB and GBSBF1 were identified using a threshold of |log2FC| ≥ 1 and *p*-adjusted < 0.05, and the data were analyzed using the R studio.

### 3.14. Statistical Analysis

Statistical analysis was performed using SPSS 21.0 (SPSS Inc., Chicago, IL, USA). The data were presented as means ± S.D. Student’s *t*-test was used to compare the two groups. For the comparison of multiple conditions, a one-way analysis of variance (ANOVA) was used, with significance difference indicated as follows: * for *p* < 0.05; ** for *p* < 0.01; *** for *p* < 0.001; and ns for no significant difference.

## 4. Discussion

The proper availability of metabolic fuels, obtained either by reducing energy consumption, increasing substrate extraction from energy stores, or both, is fundamental to fish adaptation to restricted O_2_. Fish have evolved a series of physiological and biochemical means to maintain their normal physiological activities in order to adapt to the changes in dissolved oxygen, which rely on the division and cooperation among tissues such as the liver, muscles, brain, heart, and gills [19,20,21,22,23]. The response of fish to hypoxia involves considerable changes in their behavior and physiology, including reduced swimming and food intake, air surface respiration, changes in the gill system, increased erythrocyte count, and enhanced lipid metabolism in the liver [24]. In this study, there were differences in the concentration of red blood cells and hemoglobin, as well as the gill filament longitude between GBSBF1 and BSB (Figure 1). Our results indicated that these physiological features supported the superiority of GBSBF1 strain resistance against hypoxia. Alternatively, these physiological features could also be related to HIF. In subsequent studies, we could consider exploring the possible mechanisms underlying the better hypoxia tolerance of GBSBF1 compared with BSB from these perspectives.

As an important metabolic organ, the liver is prone to hypoxia due to its high metabolic characteristics and is a major target of hypoxic stress [25]. The oxygen content in the liver is the core medium for controlling the balance of energy metabolism of the liver. In this process, the HIF signaling pathway is crucial in liver metabolism [26,27]. Under hypoxic conditions, HIF-1α induces PGK (Phosphoglycerate kinase) and LDHA (Lactate Dehydrogenase A) to regulate glycolysis [28]. The activation of HIF-1α also involves lipid metabolism or lipid synthesis and plays a key role in liver and heart metabolism [29,30,31]. Additionally, studies have reported that the activation of HIF-2α can inhibit fatty acid oxidation and lipid synthesis, increasing lipid storage capacity [32,33,34]. The loss of VHL or hypoxia causes the activation of HIF and many other genes that are involved in the regulation of cellular metabolism, which increases intracellular glucose uptake and increases glycolysis [35]. Targeted disruption of VHL in the liver increases the expression of HIF-1α and HIF-2α, and hepatic steatosis in vhl-deficient mice is caused by HIF-2α rather than HIF-1α [36]. Unlike mammals, some studies have shown that HIF-3α primarily responds to hypoxia signals and correctly activates the HIF signaling pathway in zebrafish [37,38,39]. In this study, it indicated that the primary HIF-α isoform affected in GBSBF1 was HIF-3α, aligning with previous reports in other fish [40,41]. Through omics data analysis and experimental validation, we found that the expressions of PHD2 and VHL in GBSBF1 were significantly lower than those in wild-type BSB, and their low expressions mainly affected the protein activity of HIF-3α. This helps prevent the hydroxylation of HIF-3α and ubiquitin-mediated proteasomal degradation. Consequently, the level of active HIF-3α increases, thereby contributing to the activation of the HIF signaling pathway.

As a transcription factor, HIF can attach to a number of target genes containing HREs and regulate their transcriptional expression. In this study, we chose PPAR-γ as a candidate target because there were significant differences between GBSBF1 and BSB. PPAR-γ is a member of the nuclear receptor transcription factor family, and studies have demonstrated that it is a direct target of HIF-α in mammals [42]. HIF-1α activates PPAR-γ, which activates genes involved in fatty acid uptake and glycerolipid biosynthesis. These changes increase glycolytic flux and glycolipid conversion through the glycerol-3-phosphate (G3P) pathway. The synthesis of TAG requires free fatty acids (FFAs) and the essential intermediate substance G3P. G3P can be generated by GPD1 and serves as a substrate for GPAT, the rate-limiting enzyme in the de novo synthesis of TAG [43,44]. According to studies, genes encoding GPD1 and GPAT have been found to be direct transcriptional targets of PPAR-γ in mice and humans [45,46,47]. However, in fish, particularly in *Megalobrama amblycephala*, it has not been reported whether PPAR-γ is a direct target of HIF-α or whether the genes encoding *gpd1b* (GPD1) and *gpam* (GPAT) are direct transcriptional targets of PPAR-γ. In this study, the activated HIF-3α can bind to the HRE on the PPAR-γ promoter to promote its transcription. To control the lipid metabolism and glycolysis pathways in the liver, the transcribed PPAR-γ may bind to the PPREs of the *gpd1b* and *gpam* genes, initiating the expressions of gpd1b and gpam.

The decreased PHD2 and VHL in GBSBF1 contribute to hypoxia tolerance, but the reasons for their reduction still remain to be explored. Since half of the chromosomes in GBSBF1 are derived from GBSB, we speculate that during the preparation of GBSB, factors such as ultraviolet irradiation, low-temperature shock, and gene introgression of paternal sperm may have modified the GBSB genome, leading to changes in the expression of related genes. Additionally, HIF is a broad-spectrum transcription factor that regulates a series of target genes, resulting in extensive physiological changes. We believe that the active HIF signaling pathway may have other mechanisms to help GBSBF1 adapt to the hypoxic environment. For example, mitochondria are the powerhouses of cells and are mainly responsible for generating ATP, which serves as the primary energy source for cells. Mitochondria play an important role in fatty acid metabolism, TAG lipolysis, and TAG formation. Oxygen is the core substrate for mitochondrial energy metabolism. During hypoxia, it is prone to causing the accumulation of reactive oxygen species and the conversion of metabolism. The enhanced hypoxia tolerance obtained by GBSBF1 due to the conversion of energy metabolism may be related to mitochondria. The relevant issues mentioned above will be the direction of our further research.

## 5. Conclusions

The investigation in this research indicated GBSBF1 is a new variety of blunt snout bream with enhanced hypoxia tolerance and potential for large-scale farming. The decreased expression of PHD2 and VHL in GBSBF1 leads to a significant increase in active HIF-3α protein that is not ubiquitinated and degraded. Furthermore, the activated Hif-3α positively regulates *gpd1ab* and *gpam* through PPAR-γ, increases lipid synthesis and glucose metabolism, and reduces lipolysis. The altered metabolic pattern allows GBSBF1 to better adapt to the hypoxic environment, thereby enhancing its tolerance of hypoxia.

## Data Availability

Data is contained within the article and Supplementary Materials.

## Supplementary Materials

The following supporting information can be downloaded at: https://www.mdpi.com/article/10.3390/ijms26062613/s1.
