# Supplementary material for: HIF-3α/PPAR-γ Regulates Hypoxia Tolerance by Altering Glycolysis and Lipid Synthesis in Blunt Snout Bream (Megalobrama amblycephala)
Source: Int J Mol Sci. 2025 Mar 14;26(6):2613. doi: 10.3390/ijms26062613 (PMC11942064; doi:10.3390/ijms26062613)
Supplement: Supplementary file 1 [file ijms-26-02613-s001.zip › ijms-3469907 - Supplemental Tables-author revised - 3.17.pdf]

Supplemental Tables

Table S1. Primers for qRT-PCR

Gene name	Gene annotation	Sequences (5' to 3')		GenBank NO.
fasn	fatty acid synthase	F	CGCATGTTCCTCTAGCCTG	XM_048171583.1
		R	ATAGCCATTTCCTGAGGCG	
dgat1a	diacylglycerol O-acyltransferase 1a	F	GTCTTCACCGTTCTGTTCC	XM_048203124.1
		R	GGTTTCCTGGATAGGTCAC	
ppar-γ	peroxisome proliferator-activated receptor gamma	F	CGACGACAGCGGATACAG	XM_048173304.1
		R	CGTAATGGAAGCCCGAGG	
srebf1	sterol regulatory element binding transcription factor 1	F	GTCTCTGGACACACAGGAAG	XM_048187188.1
		R	GGTTCTCCATACACGAGCA	
cpt1aa	carnitine palmitoyltransferase 1Aa (liver)	F	CTGGTGTTGGCAGGGTAC	XM_048185179.1
		R	GGCAATCCACAGACCCATG	
pparaa	peroxisome proliferator-activated receptor alpha a	F	ATCTCTACAACCATTGAAGA	XM_048154865.1
		R	CACACAGAGGGCTGTCCAAA	
slc27a1b	solute carrier family 27 member 1b	F	GACGAGGACACCATGGAG	XM_048195067.1
		R	GCCTCTTGATTCGCGTAGC	
acaa2	acetyl-CoA acyltransferase 2	F	ATCATGGGAATTGGGCCTGT	XM_048208885.1
		R	CCAAAGCTTTAGCCACTGCC	
pgm1	phosphoglucomutase 1	F	TCGGTGCCAACATTACTTCCA	XM_048199912.1
		R	AGCTTTGGCCACCTTGTCTAA	
adh5	alcohol dehydrogenase 5	F	TCCGAATACACTGTGGTGGC	XM_048176147.1
		R	AGCCGGCTTCAACTTTAGCA	
g6pc1a.1	glucose-6-phosphatase catalytic subunit 1a, tandem duplicate 1	F	GGATCTCCACACCACGTTCT	XM_048171060.1
		R	TCACCGAAAAGCACCCACTT	
pgm2	phosphoglucomutase 2	F	GGGGTCCCAGTCTACCTCTT	XM_048197502.1
		R	CCATTTGCCCAGTACACCTTG	
hif-1α (hif1aa)	hypoxia inducible factor 1 subunit alpha a	F	GCAATAAGGCTGGGGTCTGT	XM_048205311.1
		R	CGACATCAGATGGCAGCAAC	
hif-3α (hif1al)	hypoxia inducible factor 1 subunit alpha, like	F	GCCGGTCTGCCTATGAGTTT	XM_048160905.1
		R	CCTCGGGTTGAGAAGTCCTG	
hif-2α(epas1a)	endothelial PAS domain protein 1a	F	CCCGGTACCAGTATGACAGC	XM_048170134.1
		R	TGTCAGTTCCACCTGCGTAA	
Pdh2(egln1a)	egl-9 family hypoxia-inducible factor 1a	F	GACAACCCAAATGGCGATGG	XM_048203945.1
		R	GGTTCGATGTCCGCAAACTG	
vhl	von Hippel-Lindau tumor suppressor	F	TCGCTGTCTGCAAGTTGTGA	XM_048199441.1
		R	CTGTTCCACTCGCTGGCTAA	
β-actin	actin, beta 2	F	GATTCGCTGGAGATGATGCTC	XM_048192430.1
		R	CGATGGGGTACTTCAGGGTC	

Table S2. Antibodies used in this study.

Antibody	Vendor	Catalog no.	Working dilution and MW
HIF-1α	HuaBio	ER1802-41	1:1000 (WB);120KDa
HIF-2α	HuaBio	ET7107-32	1:1000 (WB);110KDa
HIF-3α	HuaBio	ER1910-61	1:1000 (WB);IHC(1:500);74 kDa
PHD2	HuaBio	R1510-40	1:1000 (WB);IHC(1:200);46 kDa
pVHL	Thermo	PA5-13488	1:1000 (WB);IHC(1;1000);24 kDa
β-ACTIN	Proteintech	66009-1-Ig	1:5000 (WB);42KDa
