# Supplementary material for: HIF-3α/PPAR-γ Regulates Hypoxia Tolerance by Altering Glycolysis and Lipid Synthesis in Blunt Snout Bream (Megalobrama amblycephala)
Source: Int J Mol Sci. 2025 Mar 14;26(6):2613. doi: 10.3390/ijms26062613 (PMC11942064; doi:10.3390/ijms26062613)
Supplement: Supplementary file 1 [file ijms-26-02613-s001.zip › supplement-figs-IJMS.pdf]

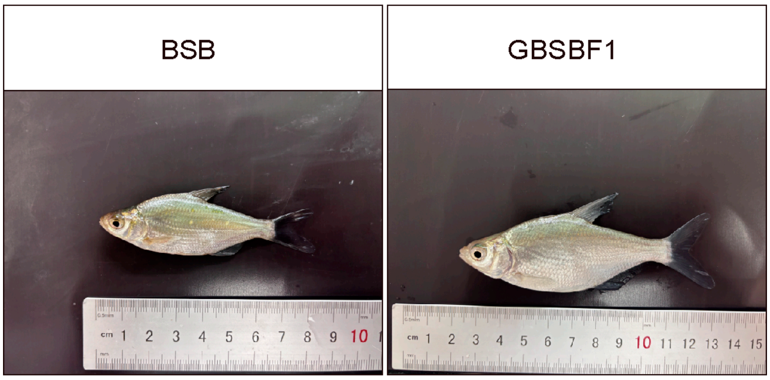

Supplemental Figure S1. Morphological observation of GBSBF1 and BSB.

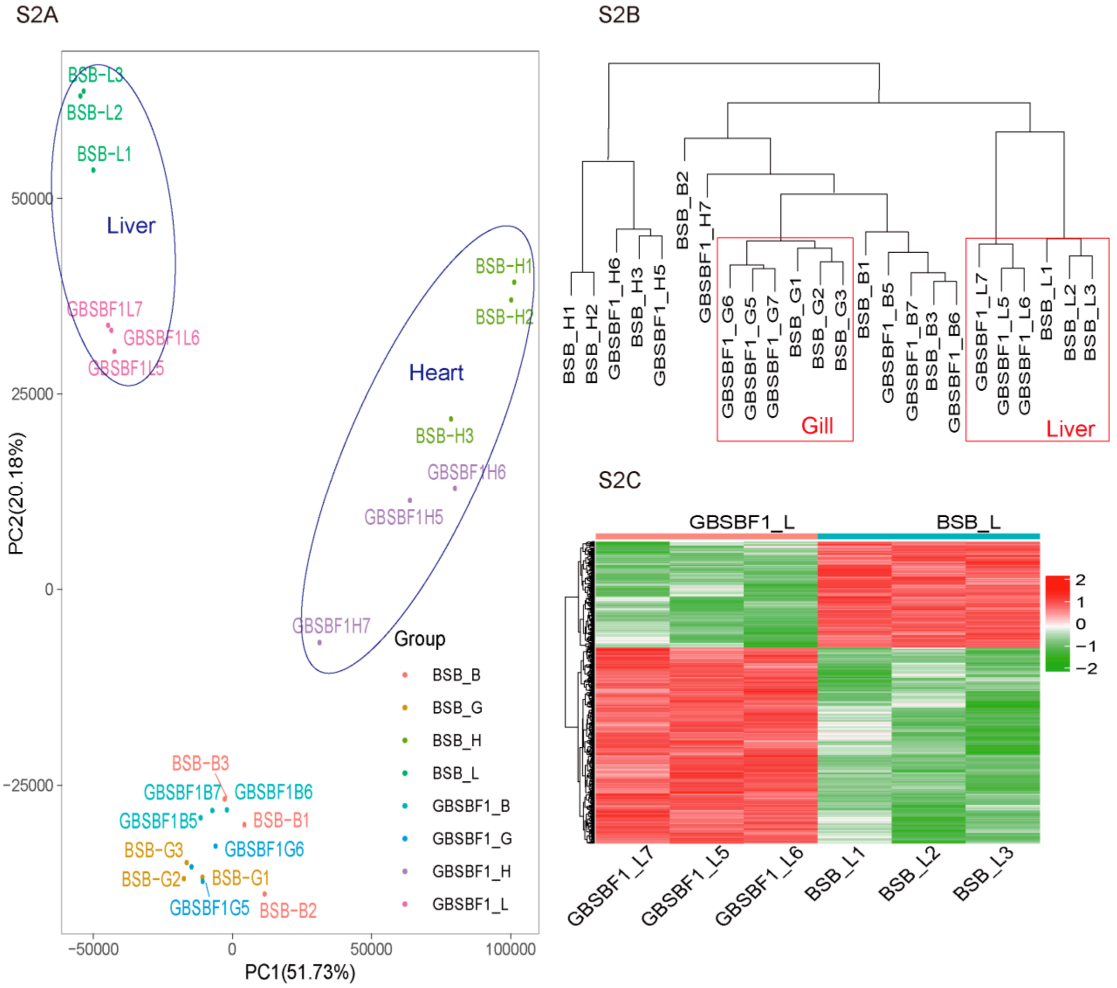

Supplemental Figure S2. RNA-seq datas of tissue samples of BSB and GBSBF1. (A) PCA analysis of RNA-seq data (B stands for Brain; L stands for Liver; G stands for Gills; H stands for Heart). (B) Cluster analysis of RNA-Seq data. (C) The heatmap shows all differentially expressed genes (DEGs) in RNA-seq.

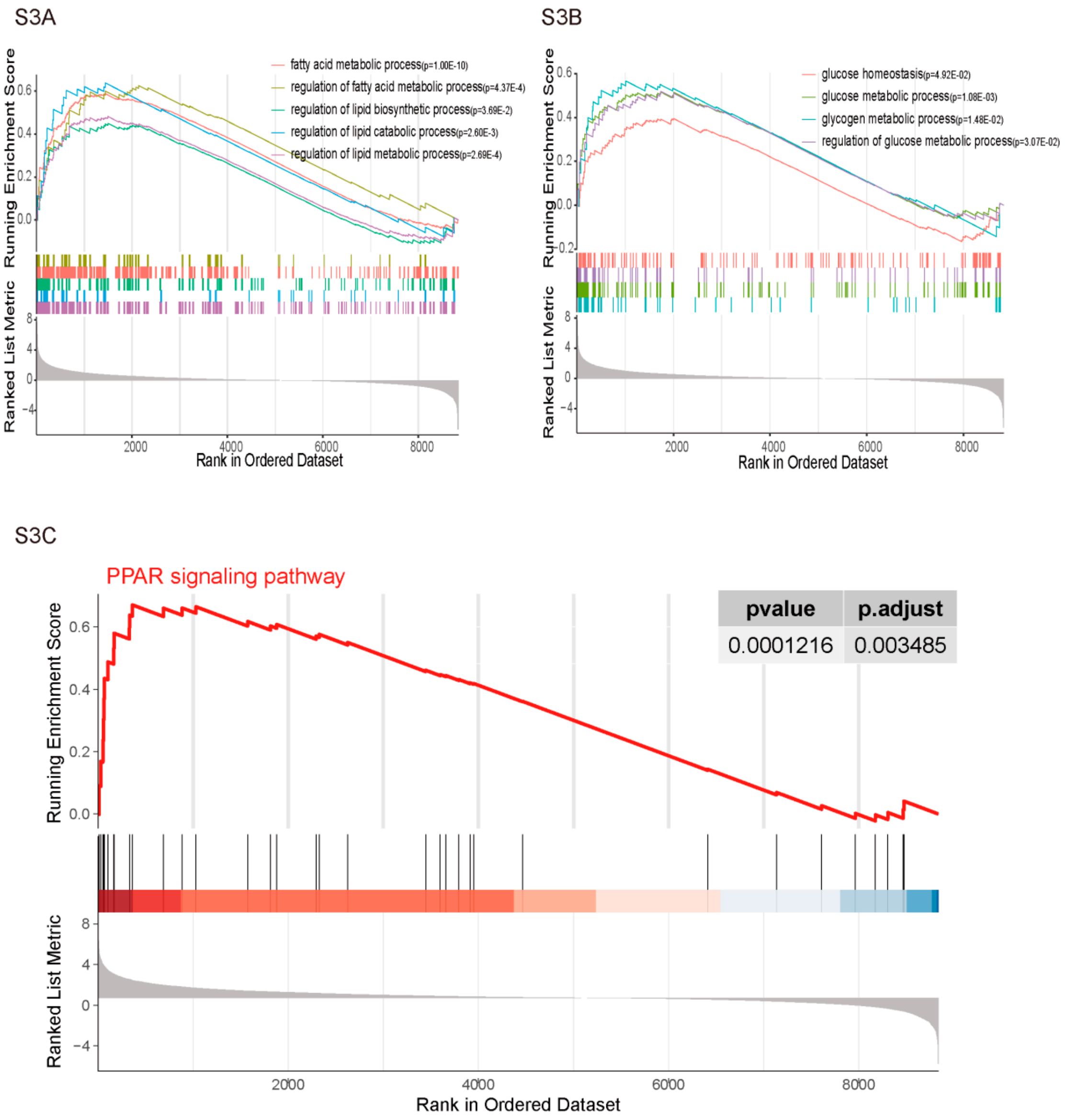

Supplemental Figure S3. GSEA analysis of biological processes (BP) in GO pathways. (A) The GO pathways (BP) directly associated with lipid metabolism showed significant differences in their expressions among different liver samples. (B) The GO pathways (BP) directly associated with glucose metabolism showed significant differences in their expressions among different liver samples. (C) PPAR signal pathway in GSEA analysis showed significant differences in their expressions among different liver samples.

.
